# Challenges of controlling sleeping sickness in areas of violent conflict: experience in the Democratic Republic of Congo

**DOI:** 10.1186/1752-1505-5-7

**Published:** 2011-05-26

**Authors:** Jacqueline Tong, Olaf Valverde, Claude Mahoudeau, Oliver Yun, François Chappuis

**Affiliations:** 1 Médecins Sans Frontières, Rue de Lausanne 78, 1211 Geneva, Switzerland; 2 Drugs for Neglected Diseases initiative, 15 Chemin Louis Dunant, 1202 Geneva, Switzerland; 3 Geneva University Hospitals, 4 rue Gabrielle-Perret-Gentil, 1211 Geneva 14, Switzerland

## Abstract

**Background:**

Human African trypanosomiasis (HAT), or sleeping sickness, is a fatal neglected tropical disease if left untreated. HAT primarily affects people living in rural sub-Saharan Africa, often in regions afflicted by violent conflict. Screening and treatment of HAT is complex and resource-intensive, and especially difficult in insecure, resource-constrained settings. The country with the highest endemicity of HAT is the Democratic Republic of Congo (DRC), which has a number of foci of high disease prevalence. We present here the challenges of carrying out HAT control programmes in general and in a conflict-affected region of DRC. We discuss the difficulties of measuring disease burden, medical care complexities, waning international support, and research and development barriers for HAT.

**Discussion:**

In 2007, Médecins Sans Frontières (MSF) began screening for HAT in the Haut-Uélé and Bas-Uélé districts of Orientale Province in northeastern DRC, an area of high prevalence affected by armed conflict. Through early 2009, HAT prevalence rate of 3.4% was found, reaching 10% in some villages. More than 46,000 patients were screened and 1,570 treated for HAT during this time. In March 2009, two treatment centres were forced to close due to insecurity, disrupting patient treatment, follow-up, and transmission-control efforts. One project was reopened in December 2009 when the security situation improved, and another in late 2010 based on concerns that population displacement might reactivate historic foci. In all of 2010, 770 patients were treated at these sites, despite a limited geographical range of action for the mobile teams.

**Summary:**

In conflict settings where HAT is prevalent, targeted medical interventions are needed to provide care to the patients caught in these areas. Strategies of integrating care into existing health systems may be unfeasible since such infrastructure is often absent in resource-poor contexts. HAT care in conflict areas must balance logistical and medical capacity with security considerations, and community networks and international-response coordination should be maintained. Research and development for less complicated, field-adapted tools for diagnosis and treatment, and international support for funding and program implementation, are urgently needed to facilitate HAT control in these remote and insecure areas.

## Background

Significant progress has been made towards the elimination of human African trypanosomiasis (HAT; sleeping sickness), which has historically ravaged communities with serious socioeconomic impacts. HAT is now confined to specific geographic foci [1,2] characterized by remoteness and neglect, and commonly in areas of political instability and/or armed conflict, with the bulk of the known disease burden in the Democratic Republic of Congo (DRC) [3].

Sustained instability and violence have massive impacts on the health of affected populations. In DRC and elsewhere more people die of treatable diseases during conflict than they do of conflict-related injuries or casualties [4-7]. This is partly because the already poor state of health care services in these areas is further degraded to where preventable diseases requiring only basic interventions, such as malaria, measles, or diarrhoea, can run rampant. HAT caused by *Trypanosoma brucei gambiense *is a particularly problematic disease and starts to surge during conflict in endemic areas. Given the time frame for disruption of HAT control activities, evolution of the transmission cycle, and disease progression, incidence can peak several years on.

HAT develops in two stages: early, haemolymphatic (stage 1) and late, neurologic (stage 2) disease. If left untreated, stage 2 HAT progresses almost invariably to death, with very few cases escaping this by resolving or becoming a chronic carrier [8]. Screening, treatment, and management are notoriously difficult. The reasons are numerous and include the fact that HAT diagnostic tools are dated and often difficult to use in resource-limited settings. For instance, a lumbar puncture is required to establish if a person is in the late neurologic stage of the disease. Other factors include difficulties in accessing known or suspected endemic areas that are remote and/or insecure, lack of robust surveillance of old foci, and complex treatments that are labour- and resource-intensive.

Médecins Sans Frontières (MSF) has been diagnosing and treating HAT for over 25 years. Currently MSF provides HAT screening and treatment in DRC and Central African Republic (CAR) and supports Ministry of Health (MOH) activities in Uganda and South Sudan. We discuss here the operational and medical challenges of managing HAT in general and in conflict areas, focusing on one region in DRC.

## Difficulties Estimating Disease Burden of Sleeping Sickness

HAT has ravaged Africa over the last century with severe epidemics in West Africa, Kenya, Tanzania, Uganda, Nigeria, and the Congo basin [9]. By the 1960s, the disease was brought under control through vector control and mobile teams conducting active surveillance of the population, implemented by colonial powers. However, neglect and complacency led to the re-emergence of HAT in the mid-1970s, and outbreaks continued until the end of the 20^th ^century.

Recent data show that HAT has been brought under control again in certain areas owing to the cessation of large-scale conflicts, such as in Angola; substantial collaborative efforts amongst the World Health Organisation (WHO), national control programmes, and nongovernmental organisations (NGOs) using ambitious vertical programmes for active case finding and treatment; and agreements from key pharmaceutical companies (sanofi-aventis and Bayer) to provide free antitrypanosomal drugs. In May 2007, a report from a WHO consultation meeting on sustainable HAT control concluded that elimination was possible [10]. Nevertheless, elimination of HAT as a public health problem will require continuous efforts and innovative approaches [11].

**Figure 1:**
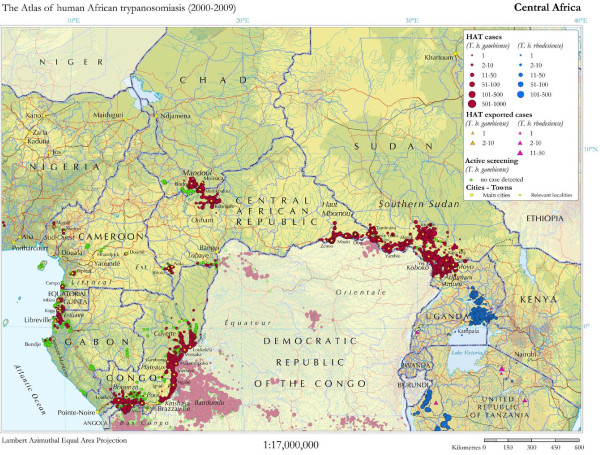
**High-prevalence HAT areas in central Africa, 2000-2009**. Source: Reproduced under open-access attribution from Simarro PP et al. *Int J Health Geogr *2010, 9:57. [12]

WHO has undertaken an ambitious global mapping exercise for HAT [12] (Figure 1), and current data show a significant decrease in reported cases over the past decade. In 1998, a high peak of 37,385 *T.b. gambiense *HAT cases was reported [13]. In 2004, a total of 17,130 cases were reported, and 9,688 in 2009 [12]. The countries with the highest incidence of new cases in 2009 are DRC with 7,183, CAR with 1,054, Chad with 510, and Sudan with 376. Over time the DRC has consistently held by far the bulk of the disease burden [12].

The number of detected cases of HAT has no doubt decreased in the last decade. However, large endemic areas escape surveillance, and most HAT patients are likely to remain undetected until these neglected foci receive attention [1]. Figures of reported cases therefore need to be treated with caution.

Many at-risk areas often struggle to provide any sort of basic health care and lack overall capacity to undertake complex diagnostics and treatment, as well as to respond with active case finding. Therefore, certain HAT hot spots remain and have in common the general problems of poverty, remoteness, instability, and insecurity (Figure 1).

## Medical Complexities of Hat Care

Providing medical care for HAT, including active screening, diagnosis, treatment, and follow-up, is a daunting challenge in remote, resource-poor, and insecure settings [14]. Active screening, in which mobile teams regularly travel to remote villages to test the population, is highly important for extensive and early case detection, leading to disease control. Passive screening entails testing at fixed health sites and is mostly insufficient for HAT control as only a fraction of the *T.b. *gambiense-infected population has access to health facilities. Also, as stage 1 HAT can mimic less serious diseases, infected people often do not present for diagnosis until stage 2, prolonging their time in the community as part of the transmission cycle. Despite greatly increasing detection, active screening is more logistically complex and costly than passive surveillance.

The current tools for HAT diagnostics are dated and require specific skills, training, and equipment. To diagnose the neurological late stage of the disease, a lumbar puncture is required to identify trypanosomes and count white blood cells in the cerebrospinal fluid (CSF). Lumbar puncture is painful and uncomfortable for the patient and difficult to carry out by the practitioner, especially in resource-limited settings, and commonly cause fear and suspicion that lead to reluctance to testing. In addition, the visualisation of trypanosomes and counting of white cells in the CSF require significant laboratory skills.

Existing treatment options are complex and require a significant level of health-care capacities. Treatment of stage 1 HAT (7 days of daily intramuscular pentamidine for *T.b. gambiense *HAT) is relatively uncomplicated but still requires skilled nursing staff. For treatment of stage 2 *T.b. gambiense *HAT, the recent addition of nifurtimox-eflornithine combination therapy (NECT) to the therapeutic arsenal has been a major improvement, by reducing the length (from 2 to 1 week) and complexity (from 56 to 14 infusions) of the previously preferred treatment of eflornithine monotherapy [15-17]. However, NECT administration remains labour-intensive, requiring 7 days of infusions of eflornithine twice a day, plus 10 days of oral nifurtimox tablets 3 times a day. A minimum of 4 nurses (to cover a 24-hour shift), to give the intravenous infusions, and a doctor, to prescribe treatment and manage potential adverse events, are required.

According to WHO recommendations, 24 months of follow-up with control visits every 6 months are required to establish HAT cure in a patient [18]. Such long follow-up is difficult to perform in resource-limited settings. As with surveillance, patients often cannot reach health facilities, and tracking patients after they have been treated and left the health centre is problematic. Other factors such as population displacements and fear of lumbar punctures also negatively affect patient follow-up care.

## Challenges of Hat Control in Conflict Zones

The constraints of complicated diagnosis and complex treatment and follow-up are compounded by the remote, rural locations in which HAT is prevalent, areas that are difficult to access and often experience violent conflict or political instability (Table 1). This poses a serious challenge because to effectively treat cases and lower prevalence in an affected area, the ability to travel and actively find cases with mobile teams is crucial. Although passive case finding is important and has an impact on overall mortality [19], more cases of late-stage than early haemolymphatic stage disease are typically found, and passive screening alone is insufficient to decrease transmission close to the elimination threshold. If medical teams cannot reach patients or people are unable to travel to health sites due to insecurity or conflict, patient care and disease control are severely impaired.

**Table 1:** Specific challenges of HAT control in conflict zones

• Conflict-afflicted areas are often already remote with minimal (if any) health infrastructures and limited numbers of trained medical staff, and their often precarious state is further eroded by insecurity.
• Insecurity often hinders active case-finding activities since mobile teams are often restricted in their travel.

• Populations often move, hampering treatment provision and post-treatment monitoring and follow-up.

• Population movements can also trigger new foci or reactivate old ones.

• Community awareness and support are important factors for effective screening and treatment. Population displacement due to insecurity can rupture community networks.

• Direct attacks of treatment centres or transport trucks can lead to programme interruption or cessation, withdrawal of supporting international NGOs and key national staff, or disruption of logistic support.

• Difficult diagnosis, complex treatment, and long follow-up are especially challenging in conflict situations, because of the high technical skills and continuity of service required.

Post-treatment follow-up of patients is deeply disrupted in conflict zones. The negative impact of low attendance rate to follow-up visits on HAT control would depend on the efficacy of treatment administered. The rate of definite cure is high in stage 1 patients treated with pentamidine and appears to be high in stage 2 patients treated with NECT [15]. If the latter is confirmed in ongoing studies and pharmacovigilance activities on larger numbers of patients, the relevance of systematic patient follow-up would become questionable. Allocating scarce existing resources to other control activities may be more cost-effective.

Mobilising the community to raise awareness and gain support for screening and treatment is also critical. During periods of political instability and conflict, the community can be stressed, people may be displaced, and the leadership and organisation of community life is often disrupted. Therefore, although highly difficult during times of conflict, establishing and maintaining the networks necessary for community support of an effective medical programme is important.

### HAT and Conflict in the DRC

All of the constraints and challenges of HAT treatment in resource-poor conflict zones can be found in the Haut-Uélé and Bas-Uélé areas of the Orientale Province of northeastern DRC. No HAT activities had been undertaken for over three decades before 2007, mainly because of the remoteness of the areas [1]. These areas border others with a history of HAT in CAR and South Sudan.

**Figure 2:**
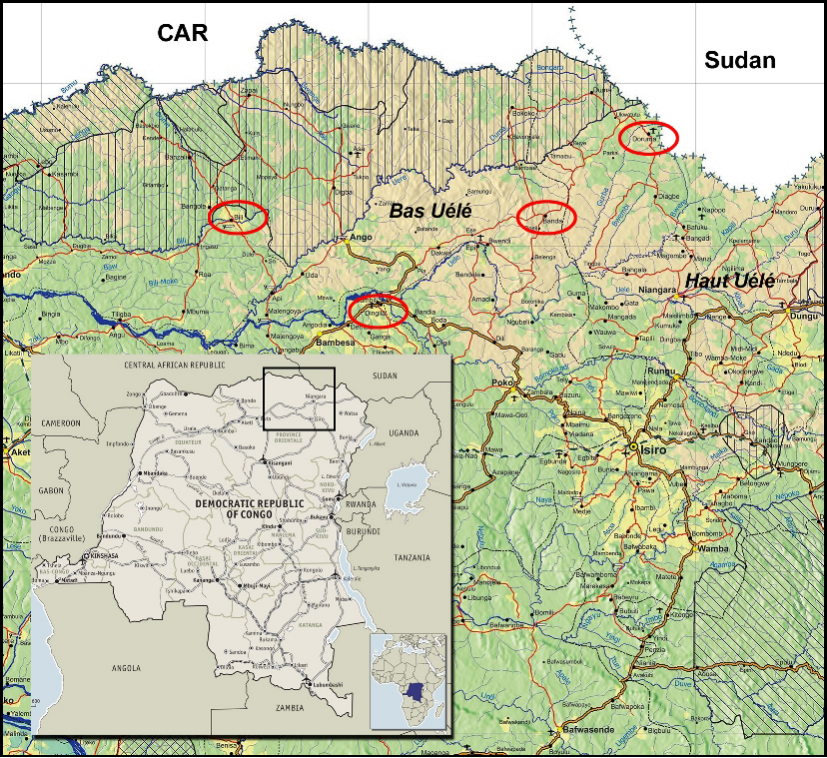
**MSF HAT programme sites in Orientale Province, DRC, December 2010**.

In mid-2007, MSF launched projects to detect and treat HAT in the zones de santé (administrative districts) of Doruma, Ango, and Bili in Orientale Province (Figure 2). From June 2007 to March 2009, MSF found areas of high infection, and 3.4% (1,570) of the 46,601 people screened were positive and treated for HAT [1], with some pockets as high as 10%. A large proportion of cases were diagnosed in the early stage (60%), indicating intense transmission. These rates are worrisome and amongst the highest reported in DRC.

In early 2008, the MSF treatment centre in Bokoyo was closed for over one month because of conflict-related insecurity. From September 2008, this insecurity and violence, which had been exacerbated by joint military operations undertaken by the Congolese army together with Ugandan troops against the Lord's Resistance Army (LRA), threatened all MSF activities in the region.

In March 2009, the town of Banda was attacked, kidnappings occurred, and the MSF compound was looted. All the medical stock was taken and the referral HAT treatment centre looted. Following this, all MSF HAT projects in the area (Doruma, Ango, Bili) were suspended. The lack of trained staff in existing health structures and the complexity of HAT diagnosis and treatment prevented any emergency handover of the project to local partners. Prolonged interruption of the projects thus resulted in people not being diagnosed and treated, lack of follow-up of patients already treated, and disruption of initial control efforts, with likely subsequent deaths and increased disease transmission. Prior to suspension of activities, the geographical limits of the endemic focus had not been reached. Moreover, with the conflict came displacement, so concerns arose of HAT spreading into new areas or reactivating old foci with population movements.

In December 2009, MSF undertook an assessment of the Doruma health site, and the project was reopened there the following month, performing active screening and treatment. In all of 2010, 485 patients were treated in Doruma. Because of the ongoing conflict and insecurity, only a relatively small area (~10-km enclave) of Doruma could be covered by the mobile team. This exclusion of previous sites restricted the overall impact of HAT control efforts in the region. Active screening extended to further areas remains dependent on the ever-changing security situation.

Also in December 2009, exploratory missions of two areas in Bas-Uélé district, Dingila and Poko, were carried out. These explorations were performed because of the displacement of populations from the Ango health zone to the south, presence of HAT-transmitting tsetse flies in the region, and accessibility of the areas due to previous MSF intervention (measles vaccination campaign). A relatively high number of HAT cases were found in Dingila: 28 (4.4%) of 630 tested individuals, with most cases in early-stage disease indicating intense transmission. This finding was alarming in part because this location, south of the Uele River, had had no recent cases of HAT, but according to the local population, people in the area were treated for HAT during the 1960s. Thus, the possibility existed of rapid re-infestation of this area based on previous endemicity. A new HAT project was opened in Dingila in September 2010. Through December 2010, 365 (3%) of 12,281 people screened were diagnosed with HAT and 285 treated.

### Impacts in Bordering Areas

Political instability and conflict often cause people to flee in order to seek refuge. One consequence is that those infected by HAT are unable to access treatment or fail to obtain follow-up care. Another potential consequence is that those infected with HAT can possibly spread the disease by entering a new cycle of transmission as the parasite may thrive in previously uninfected vectors. Displaced populations in the Orientale Province of DRC are entering new regions, raising the risk of reactivating historically cleared pockets or creating new foci, as suggested (though not proven) by the case of Dingila. Due to insecurity and the challenges of responding to conflict, controlling and understanding HAT spread because of displacement is extremely difficult.

Moreover, the concerted response by the national armies to the conflict is pushing the LRA to move their activities into areas of CAR, where HAT is endemic, triggering further displacement of local populations. Haut-Uélé also borders Uganda and South Sudan, with the latter highly under-resourced and subject to sporadic conflict and political tension. Exact figures cannot yet be confirmed, but anecdotally based on MSF experience, some HAT cases in Congolese refugees from Haut-Uélé have been found in Yambio in South Sudan.

Numbers of displaced persons change often; population movements can be mercurial, and people often go unregistered. For 2009, the United Nations High Commissioner for Refugees (UNHCR) reported that 20,899 refugees from DRC entered CAR and 19,709 entered Sudan [20]. These figures do not give a breakdown of where in DRC people have been displaced from, but a significant number are expected to have originated from the conflict in the Ueles. Effective response across borders and amongst refugees needs coordination between the respective national health authorities, and with UNHCR, which poses a challenge if capacities to carry out basic health care activities on a national level are lacking.

## Research and Development Hurdles

Research and development (R&D) for new, simpler diagnostic tools and treatments for HAT are urgently needed to eliminate the disease or at least facilitate its control. This requires setting up and performing clinical trials in HAT-endemic settings, which could include post-conflict areas. The presence of the disease in remote and unstable settings brings the challenge of feasibility of conducting clinical trials based on Good Clinical Practice (GCP) guidelines in such resource-limited contexts. The follow-up required to declare disease cure (up to 24 months), with control visits every 6 months after the end of treatment [18], can only be carried out if a long-term, stable environment is available at clinical research sites. A recent study on possible early surrogate markers of cure showed a diagnostic algorithm that could reduce the follow-up to 6 months in most patients and to 12 months in those showing doubtful results at 6-month visit, but it has not yet been formally validated [21].

The steady decline in reported HAT cases, which is a promising outcome in itself, is an additional challenge to conduct adequately powered clinical trials. On the one hand, the number of patients available to be enrolled in clinical trials is decreasing. On the other hand, the places with no control of the disease, ie, the places where the number of patients is not decreasing, are not available for research unless a long-term change in security conditions takes place.

These barriers though difficult may be overcome with careful setup of trials, including thorough assessment of local prevalence, reinforced active search activities, multiple trial sites, longer recruitment periods, and active follow-up to avoid patient loss. These activities however add to the burden of implementing clinical trials in poorly resourced settings and undoubtedly increase costs.

The Drugs for Neglected Diseases *initiative *(DND*i*) is monitoring the epidemiological evolution and political environment of HAT-endemic countries to proceed with field trials of new drug candidates in clinical development. Fexinidazole, one of these drug candidates, is now in the final stages of a Phase I clinical trial and will soon be studied in DRC [22,23]. Other oral compounds are being developed and are part of the DND*i *pipeline for HAT [24].

## Insufficient International Support and Funding

Given the successes to date of the fight against HAT, the danger exists of health authorities in affected regions downgrading HAT from "neglected" to simply ignored [25]. This attitude could extend to donors and policymakers at the international level. The decline in numbers could potentially give a reason for further disinvestment in HAT treatment programming. Minimally, where funding support is sustained, the trend may continue of integration of HAT activities in areas where it is neither feasible nor appropriate.

Solutions for providing HAT care in conflict settings thus require sustained international support from field-programme implementers and donors [11]. In this respect, a current looming obstacle is the planned reorientation and disinvestment in HAT-specific projects by a major donor for HAT programming in DRC, which carries the bulk of the known case burden. The Belgian Development Agency (BTC-CTB) has commendably been one of the major funders for the fight against HAT in DRC, and moreover, where most governmental donors have withdrawn completely, they remain one of the most aware and engaged. However, they now plan to adjust their approach and work more towards integrating HAT response projects into existing health structures, which raises concerns [26].

This shift is in line with the current international-community trend of focusing on integration of neglected tropical disease (NTD) surveillance and response into existing health structures. For HAT this poses significant difficulties: in most areas where HAT is highly prevalent, primary health care facilities are often lacking and thus nothing exists into which to integrate the complex diagnostic and treatment procedures. Integration may indeed be the solution for other NTDs, and even for HAT in settings where health care infrastructure is in place and functioning, but for HAT in resource-poor and conflict areas, specific resources for surveillance and programming are still needed.

The research funding arena has recently seen an increase between 2008 and 2009 of 34.7%, up to $46.4 million. Still, more than half of this is directed to basic research, with only one-third to new drugs and 7% to new diagnostics. As new drugs come to the door of clinical trials, further funds will be needed to bring them to the patients [27].

## Summary

History has taught us the consequences of allowing declines in disease surveillance and treatment capacity for HAT. Unchecked and untreated HAT in conflict areas, acutely exemplified by the Orientale Province of DRC, poses a significant public health threat that can extend to other regions. Results from recent research have shown that the peak incidence of HAT shows a lag time of 10 years from the start of conflict events. If this observation holds true in the case of the conflict in the DRC, we can expect more troubling prevalence rates before we see a decline [28].

The key components of HAT care and control--active screening, diagnosis, treatment, and follow-up--are a challenge to implement in endemic regions, more so in areas of violent conflict. But providing such HAT care is not impossible, as evidenced by our intervention experience in DRC. With committed resources and will, HAT patients can be accessed and treated in conflict areas, while taking into consideration security conditions and medical and logistical capabilities.

In conflict settings of high HAT prevalence, strategies of integrated care programs may not be feasible due to lack of infrastructure and security, and thus targeted (vertical or similar) medical interventions are needed. Maintaining community networks and coordinating international and national responses across regions and borders are important for reaching patients cut off from care and dealing with displacement. Further R&D is urgently required for developing new diagnostic tools and treatments for simpler, field-adapted therapy. Finally, international support for funding and program implementation must be ramped up to increase and improve HAT control in high-prevalence foci. Policymakers and donors, as well as researchers and health care workers, must be informed about the nature and specificity of this killer disease and the need for targeted interventions. In particular for people in DRC, HAT remains a serious health issue requiring ongoing collaborative work for any chance at elimination. Given the scope of the problem, without such efforts HAT could resurge with devastating public health and socioeconomic consequences.

## Competing interests

The authors declare that they have no competing interests.

## Authors' contributions

FC, OV, and CM have made substantial contributions to concept and design and data acquisition, analysis, and interpretation. OY and JT drafted the manuscript and revised it critically. All authors have read and approved the final version of the manuscript.
